# Somatosensory processing in long COVID fatigue and its relations with physiological and psychological factors

**DOI:** 10.1113/EP091988

**Published:** 2024-08-06

**Authors:** Bethan Thomas, Rachael Pattinson, Christine Bundy, Jennifer L. Davies

**Affiliations:** ^1^ School of Healthcare Sciences Cardiff University Cardiff UK; ^2^ School of Dentistry Cardiff University Cardiff UK

**Keywords:** autonomic nervous system, fatigue, long COVID, physiology, psychology, sensory processing, somatosensory system

## Abstract

Fatigue is prevalent amongst people with long COVID, but is poorly understood. The sensory attenuation framework proposes that impairments in sensory processing lead to heightened perception of effort, driving fatigue. This study aims to investigate the role of somatosensory processing impairments in long COVID fatigue and quantify how sensory processing relates to other prominent symptoms of long COVID including autonomic dysfunction, mood and illness beliefs in driving the experience of fatigue. We will recruit 44 individuals with long COVID fatigue and 44 individuals with neither long COVID nor fatigue (controls). Our primary objective is to compare baseline somatosensory processing between individuals with long COVID fatigue and controls. Additionally, we will explore the associations between somatosensory processing, fatigability and the level of fatigue induced by cognitive and physical exertion. Due to the complex nature of fatigue, we will also investigate how long COVID, state fatigue, perceived effort, mood, illness beliefs, autonomic symptoms and autonomic nervous system function interact to predict trait fatigue. This comprehensive investigation aims to elucidate how sensory processing and other prominent symptoms interact to impact the experience of fatigue.

## 1 INTRODUCTION

Long COVID is a multi‐system condition in which individuals experience persistent symptoms 12 weeks following acute COVID‐19 infection (Crook et al., 2021; National Institute for Health & Care Excellence, 2020). Fatigue is one of the most common and debilitating symptoms reported in long COVID (Joli et al., 2022), causing a significant impact on individuals, employers and healthcare systems (Townsend et al., 2020).

Fatigue is an experience of symptoms that can only be measured through self‐reporting. It is a complex phenomenon that is likely to involve overlap of physiological and psychological factors (Matura et al., 2018). Although difficult to define (Kluger et al., 2013), there has been recent consensus within another clinical condition that fatigue is a ‘range of symptoms from mild subjective feelings of tiredness to an overwhelming debilitating, and sustained sense of exhaustion that likely decreases one's ability to execute daily activities and function normally in family or social roles’ (Maxwell et al., 2024). In this study we use a multi‐framework approach to explore the complexity of potential mechanisms and drivers of long COVID fatigue. We specifically focus on the sensory attenuation model of fatigue, the role of autonomic dysfunction, the common‐sense model of self‐regulation, and the mood updating model to explore the complex interactions between physiological and psychological factors in long COVID fatigue.

The sensory attenuation model of fatigue (Kuppuswamy, 2022) proposes that impaired sensory processing increases effort perception, driving fatigue (de Doncker et al., 2018; Kuppuswamy, 2017). Somatosensory deficits exist across clinical conditions that involve fatigue (Jamali et al., 2017; Kessner et al., 2016), and somatosensory processing has been linked to fatigue (Ito et al., 2022). Sensory changes are common in long COVID (Trott et al., 2022), but the sensory attenuation model of fatigue has not been fully explored in this population.

There are two similar, but distinct, phenomena involved in somatosensory processing (Kilteni & Ehrsson, 2022). Attenuation is the suppression of somatosensory input that arises from one's own movement (reafferent input) in comparison to somatosensory input that arises from external sources (exafferent input). Gating is the suppression of externally generated somatosensory input during voluntary movement in comparison to when at rest. Somatosensory gating has been reported not to differ between individuals without long COVID and those reporting long COVID fatigue that had a moderate‐to‐severe impact on daily living (Baker et al., 2023). Previous studies in clinical conditions that experience fatigue have focused on somatosensory attenuation (Parthasharathy et al., 2022; Wolpe et al., 2018), but this has not been studied in long COVID. In this study we aim to replicate the Baker et al. (2023) finding that somatosensory gating is not different in individuals with and without long COVID fatigue, and extend this by asking whether somatosensory attenuation is different between individuals with and without long COVID fatigue (Research Question 1).

Fatigue can be considered as both a stable and enduring characteristic, reflecting how a person feels over weeks or months, and an instantaneous experience, reflecting how a person feels at a given moment. These concepts are referred to as trait and state fatigue, respectively. Although somatosensory processing has been studied in relation to trait fatigue (Baker et al., 2023; de Doncker et al., 2021), it is not well studied in relation to state fatigue. Definitions and measures of fatigue in long COVID cover different dimensions, such as physical, cognitive, emotional, psychosocial, and general fatigue and post exertional malaise (Thomas et al., 2024 [Unpublished raw data]). It is unclear if different dimensions share the same underlying mechanisms. Here we focus on state fatigue in two of these dimensions—physical and cognitive. We will quantify relations between somatosensory processing and state fatigue induced by both a cognitive and a physical task (Research Question 2) and quantify how these are related to trait fatigue (Research Question 4). This will add to our understanding of the relations between and mechanisms underlying different dimensions of state fatigue and trait fatigue in long COVID.

The sensation of fatigue may be independent from objective measures of performance (Bailey et al., 2007; Krupp & Elkins, 2000; Lou et al., 2003). For example, Fietsam et al. (2023) found that individuals with long COVID reported more fatigue than individuals without long COVID but had similar levels of performance decline on an isokinetic fatigue task. The decline in an objective measure of performance over a discrete period of time is termed performance fatigability (Enoka et al., 2021). Understanding the association between fatigue and fatigability has been identified as an important goal for clinical research (Kluger et al., 2013). We will address this in our second research question by quantifying whether the change in performance over the cognitive and physical tasks (performance fatigability) is related to state fatigue induced by that task, whether this moderates the relation between somatosensory processing and state fatigue, and whether this is impacted by long COVID (Research Question 2).

**FIGURE 1:**
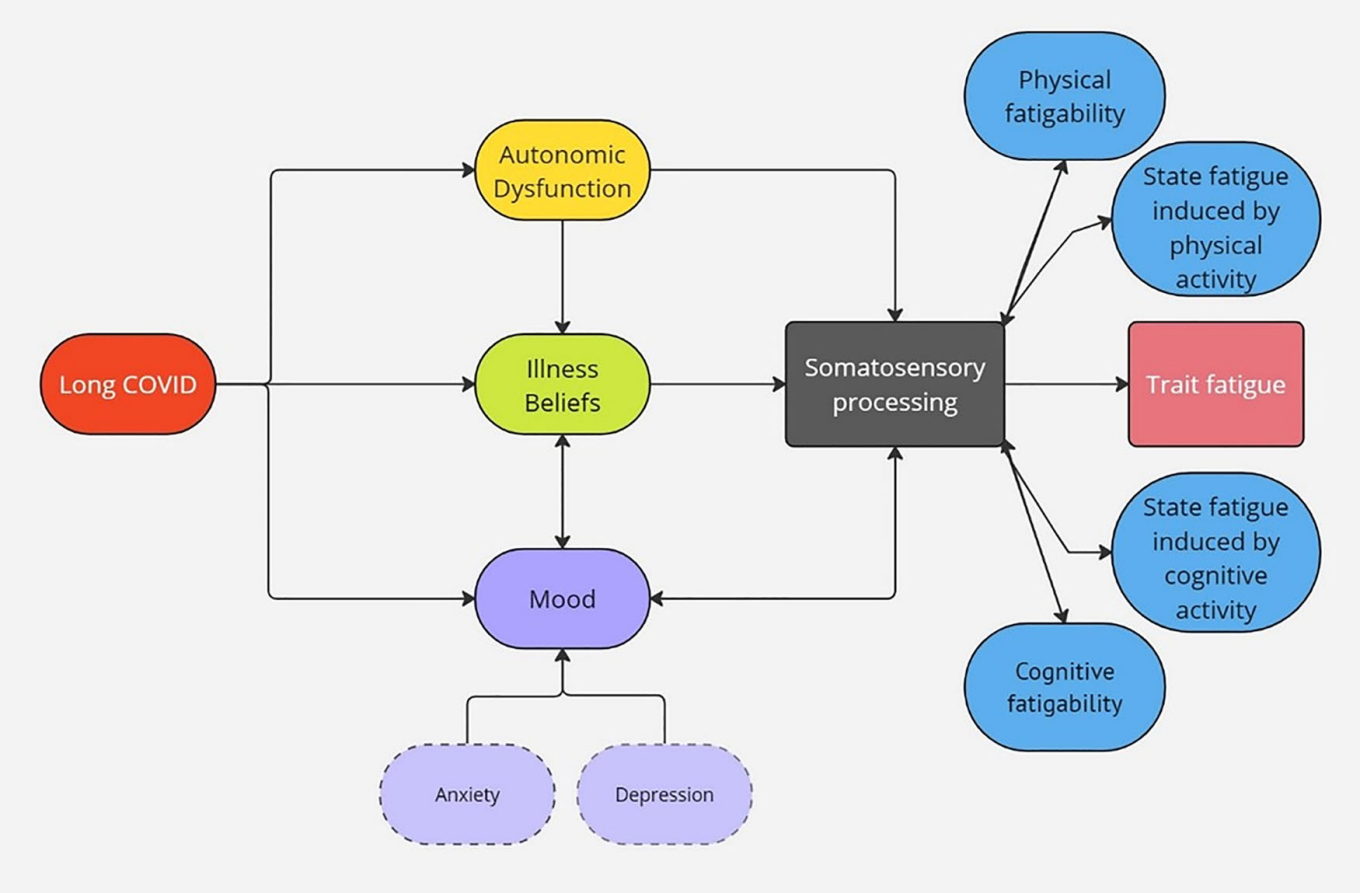
Concept of interactions. Schematic illustration indicating the proposed relations between autonomic dysfunction, illness beliefs, mood (anxiety and depression) and somatosensory processing. We postulate that each of these factors (autonomic dysfunction, illness beliefs, mood) will impact fatigue via an impact on somatosensory processing. We also propose that fatigability and acute (state) changes in fatigue will have a bidirectional relation with somatosensory processing.

In addition to understanding if somatosensory processing predicts fatigue, it is also of relevance to ask if fatigue impacts somatosensory processing (bidirectional arrows in Figure 1, blue boxes). This would potentially indicate that activities that increase fatigue and cause performance fatigability, could lead to disrupted somatosensory processing, further increasing the fatigue and fatigability. This will be addressed in our third research question, where we ask if the change in somatosensory attenuation (Research Question 3i) and somatosensory gating (Research Question 3ii) from pre‐ to post‐task is predicted by the change in state fatigue from pre to post task, fatigability during the task, population (long COVID/controls) and task (cognitive/physical).

In conditions such as depression, autonomic dysfunction is a mediating factor in the experience of fatigue (Costa et al., 2023). Autonomic dysfunction is prevalent in long COVID (Dotan et al., 2022), potentially reflecting a disrupted balance between sympathetic and parasympathetic nervous systems (Jammoul et al., 2023). However, this has not yet been directly related to fatigue in this population. We propose that autonomic dysfunction may impact fatigue via an effect on somatosensory processing (Figure 1). No studies across any clinical conditions have explored the relations between autonomic dysfunction, somatosensory processing, and fatigue. This will be considered in Research Question 4 where we explore the role of the autonomic nervous system in trait fatigue. Additionally in exploratory analysis we will look at (i) the effect of autonomic dysfunction, on state fatigue via an effect on somatosensory processing, and (ii) the relations between autonomic dysfunction during exertion, state fatigue during exertion, and perceived effort during exertion.

The common‐sense model of self‐regulation (Leventhal et al., 2016) proposes that how an individual perceives their symptoms can be influenced by their mood, previous experiences, and beliefs. The mood updating model proposes that prior beliefs about the signals resulting from an action, as well as mood (particularly depression and anxiety), can impact perception of and response to stimuli (Clark & Watson, 2023). Depression and anxiety are a feature of long COVID (Fancourt et al., 2023), and the experience of long COVID symptoms has a profound impact on self‐identity and beliefs about illness (Callan et al., 2022). There is likely a complex interaction between illness beliefs, mood, perception of stimuli and symptom experience in long COVID that has not yet been explored. Understanding individual experiences driving symptom perception and response is essential for targeted interventions. These relations will be explored in Research Question 4, where we quantify if among individuals with long COVID, if trait fatigue is effected by the increase in state fatigue induced by a physical task, the increase in state fatigue induced by a cognitive task, mood, illness beliefs, autonomic symptoms and autonomic nervous system function.

## 2 RESEARCH QUESTIONS

Research Question 1: Are pre‐task (baseline) somatosensory attenuation (Research Question 1a) and somatosensory gating (Research Question 1b) different between individuals with long COVID fatigue compared to controls?

Research Question 2: Is state fatigue following exertion predicted by somatosensory attenuation at baseline, somatosensory gating at baseline, the presence of long COVID (long COVID/controls), and the type of task (cognitive/physical), and is performance fatigability a moderator variable in this relation? State fatigue prior to exertion will be included as a covariate in this analysis.

Research Question 3: Is the change in somatosensory attenuation (Research Question 3a) and somatosensory gating (Research Question 3b) from pre‐ to post‐task predicted by the change in state fatigue during exertion, fatigability, the presence of long COVID (long COVID/controls), and the type of task (cognitive/physical)?

Research Question 4: Among individuals with long COVID, is trait fatigue predicted by the increase in state fatigue induced by a physical task, the increase in state fatigue induced by a cognitive task, mood, illness beliefs, autonomic symptoms and autonomic nervous system function?

This study will provide the first comprehensive investigation into the role of somatosensory processing in long COVID fatigue and explore how prominent symptoms in long COVID (autonomic dysfunction, altered mood, beliefs about the meaning of symptoms) interact with somatosensory processing, fatigability and the experience of fatigue.

## 3 METHODS

### 3.1 Ethical approval

Prior to recruitment of pilot participants, this study received ethical approval from Cardiff University School of Healthcare Sciences Research Ethics Committee (REC1160). The study conforms to the standards set by the *Declaration of Helsinki*, except for being registered in a publicly accessible database. This study will conduct experiments on humans, and written informed consent will be obtained from all participants.

### 3.2 Participants and recruitment

Participants will be recruited via convenience sampling from the local community using passive recruitment methods (digital and physical posters and announcements). Recruitment will be targeted to match long COVID and control groups for age and gender. To be eligible, individuals must be between 18 and 69 years old. Somatosensory attenuation and gating change with age (Parthasharathy et al., 2022), particularly after the age of 69 years (Timar et al., 2023). This age group will therefore be excluded, to prevent age‐related effects on somatosensory attenuation and gating confounding the results. Participants must be able to speak and understand English and be able to provide informed consent. Participants will be excluded if they have any physical or psychiatric condition that would prohibit them from walking safely on a treadmill, taking part in a cognitive task or completing the full battery of assessments (Supporting information, Appendix 1).

For inclusion in the control group, individuals may have previously tested positive for COVID‐19 but must have been clear of all symptoms by 12 weeks following acute infection and must not report any ongoing fatigue. Participants in the control group must not report any underlying condition that interferes with their daily function. All inclusion and exclusion criteria will be evaluated by self‐report measurements (Supporting information, Appendix 1).

For inclusion in the long COVID group, individuals must self‐report a confirmed COVID infection (confirmed by positive lateral flow test or polymerase chain reaction test, or a clinical diagnosis) and report signs and symptoms that continued or developed 12 weeks or more after acute COVID‐19 infection (National Institute for Health & Care Excellence, 2020). As this study will focus on the impact of fatigue, individuals must report fatigue (physical, cognitive, mental, emotional, psychosocial fatigue or post‐exertional malaise) as one of their long COVID symptoms and must not have experienced any form of ongoing fatigue prior to COVID infection (Supporting information, Appendix 1).

### 3.3 Experimental procedures

Prospective participants will be provided with a participant information sheet and an opportunity to ask questions. If they wish to participate, they will be directed to an online platform (JISC) and asked to fill in a consent form. Once informed consent has been provided, the participant will be contacted to schedule the two testing sessions.

**FIGURE 2:**
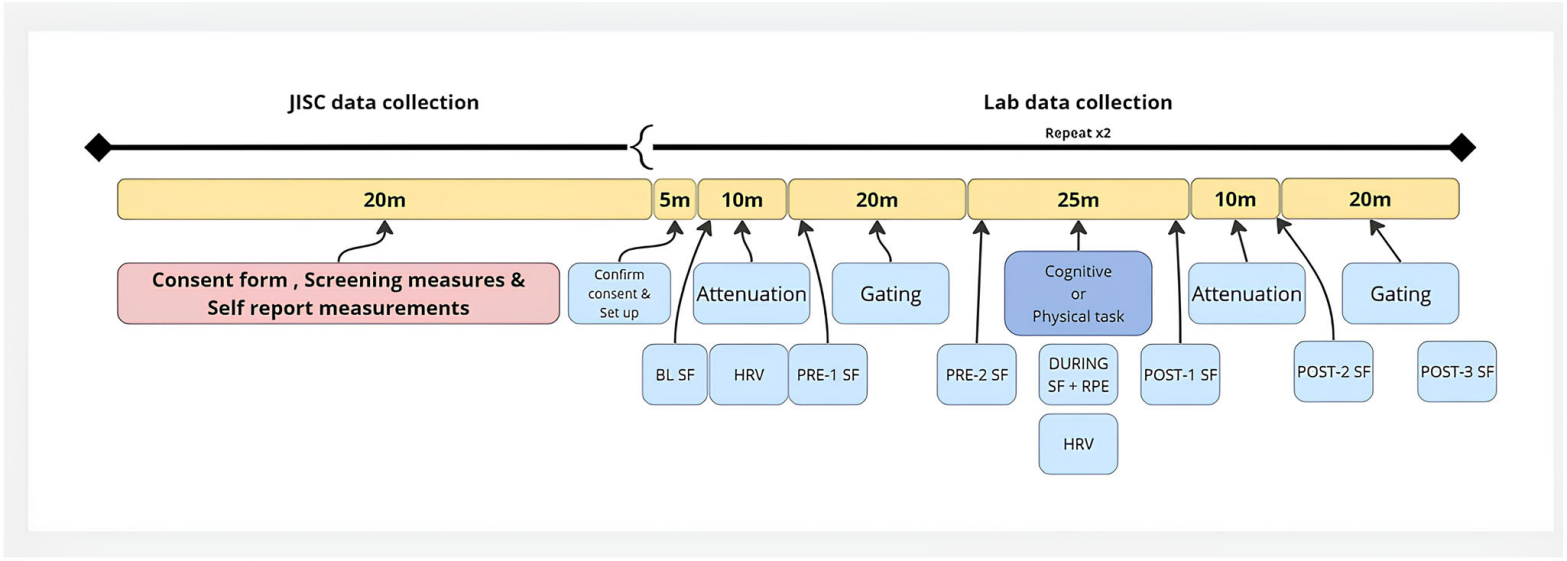
Data collection timeline. HRV, heart rate variability. JISC is a digital platform designed specifically for education and research organizations that can be used for the creation and dissemination of surveys. BL, baseline; PRE‐1, immediately after pre‐task somatosensory gating measure; PRE‐2, immediately after pre‐task somatosensory attenuation measure; POST‐1, immediately after the completion of the task (cognitive or physical); POST‐2, immediately after post‐task somatosensory gating measure; POST‐3, immediately after post‐task somatosensory attenuation measure.

A schematic illustration of the study timeline is shown in Figure 2. Prior to the testing sessions, participants will be directed to the online platform (JISC), which they are able to access from any place with internet connection to provide self‐reported measurements (Supporting information, Appendix 1) and complete a battery of validated measures (see ‘Outcome measures’). A contact email for the research team will be provided on every page of the online platform, in case of questions. Participants will be able to pause and restart this process multiple times. Pilot tests have indicated that this process will take 60 min in total.

On completion of the participant characteristics and validated measures, participants will be given a unique, randomly generated four‐digit identifier. Consent forms, which will contain the participants personal details, will be saved separately from all other data and only identified with the participants four‐digit identifier.

The protocol is split into two testing sessions. Each session is expected to take up to 90 min. The two sessions are identical, except that in one session participants perform a cognitive task and in the other session they perform a physical task. Participants will be asked to complete both sessions in a crossover design. In each session, somatosensory processing (attenuation and gating) will be measured pre‐ and post‐task (physical or cognitive). The order of the two sessions will be randomized using the command randi([1 2]) in MATLAB software version 23.2 (MathWorks Inc., Natick, MA, USA), which will be run on the morning of the first testing session. A value of ‘1’ indicates that cognitive task will be performed first and ‘2’ indicates that the physical task will be performed first. Participants will be asked to return on a separate day for each session.

Upon arrival at each testing session, the participant will be re‐consented to ensure that they are still happy to participate and will then be prepared for data collection. The measure of somatosensory gating requires activity of the first dorsal interosseus muscle to be monitored in real time (see ‘Outcome measures’). To achieve this, the skin over the first dorsal interosseus muscle will be prepared using a mild abrasive gel (NuPrep, Weaver and company, Aurora, CO, USA) and a Trigno Quattro Sensor (Delsys Europe, Manchester, UK) will be placed over the muscle belly parallel to the radial border of the second metacarpal, just proximal to the junction between the muscle and distal tendon (Zijdewind et al., 1995). The reference sensor will be placed on the forearm. Heart rate will be measured in a similar way to ECG, using a surface electrode placed on the skin over the chest (Delsys Europe, Manchester, UK).

Pre‐task measures (somatosensory gating, somatosensory attenuation, state fatigue) will be evaluated (see ‘Outcome measures’ and Figure 2). Following this, the participant will be asked to perform the task (cognitive or physical, depending on the session). Immediately following the task, post‐task measures (somatosensory gating, somatosensory attenuation, state fatigue) will be evaluated (see ‘Outcome measures’ and Figure 2).

#### 3.3.1 Cognitive task

The cognitive task is based on that developed by Hassan et al. (2024). It consists of a battery of four different cognitive tasks, each designed to challenge a different aspect of executive function: A‐X Continuous Performance Test (CPT), n‐back, mental rotation task, and visual search task. The AX‐CPT was chosen as the task in which to measure task performance, and therefore it will be presented first and last as in Hassan et al. (2024). The remaining tasks were presented in a pseudorandom order as designed in Hassan et al. (2024), where the tasks did not repeat back‐to‐back; the time between tasks being repeated was maximized; and the time between tasks being repeated was similar between the different tasks, so that if any order effect was present, it would be the same for all participants.

**FIGURE 3:**
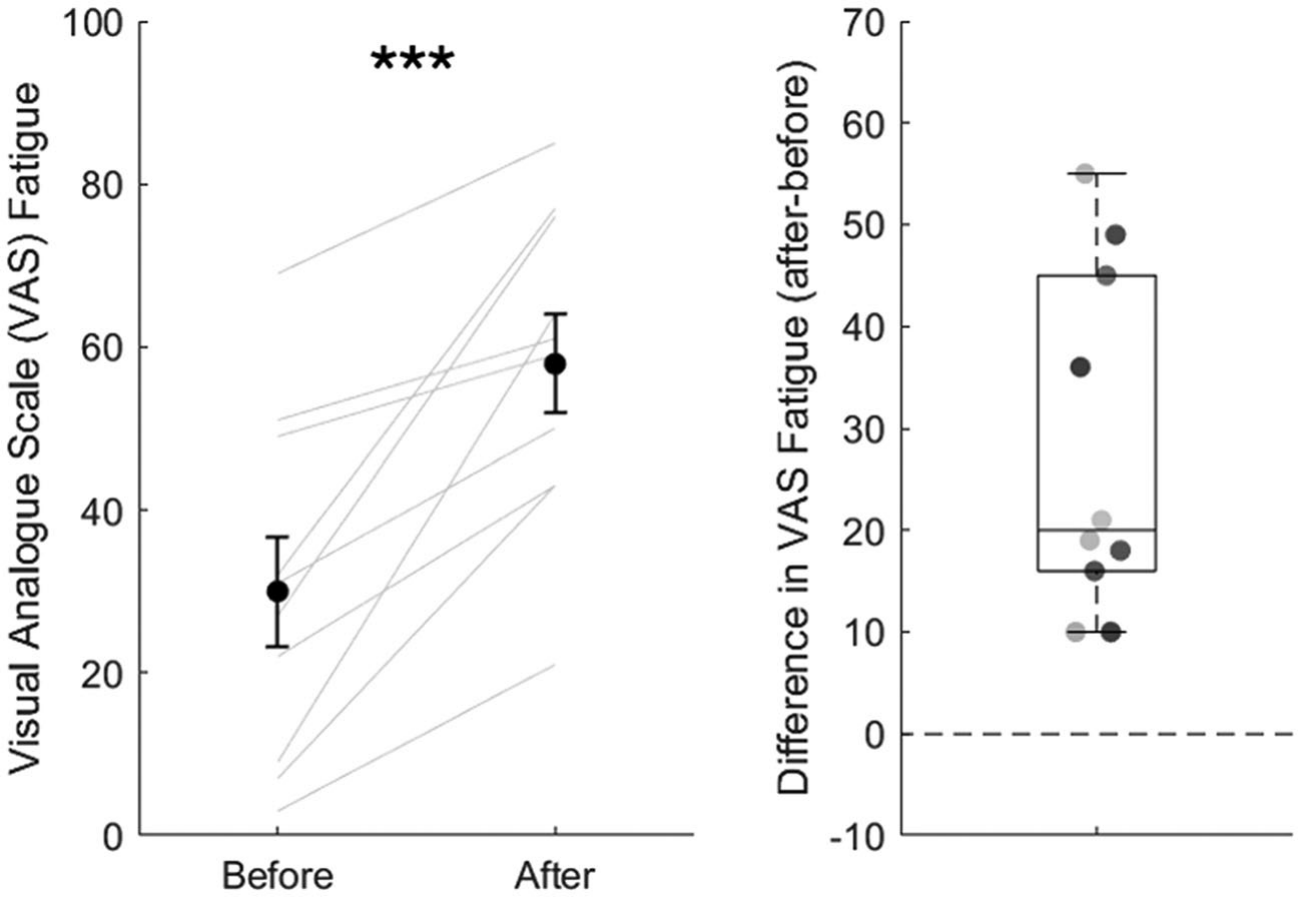
VAS‐F scores. (a) Before and after completing the cognitive fatigue task battery. Black circles represent the mean score. Error bars show ±1 standard error. Light grey lines show individual scores. (b) Difference before and after completing the cognitive fatigue task battery. Values above the dotted line represent an increase in fatigue. Statistical significance shown: ***P < 0.001. Unpublished data from Corfield and Davies (2024).

**FIGURE 4:**
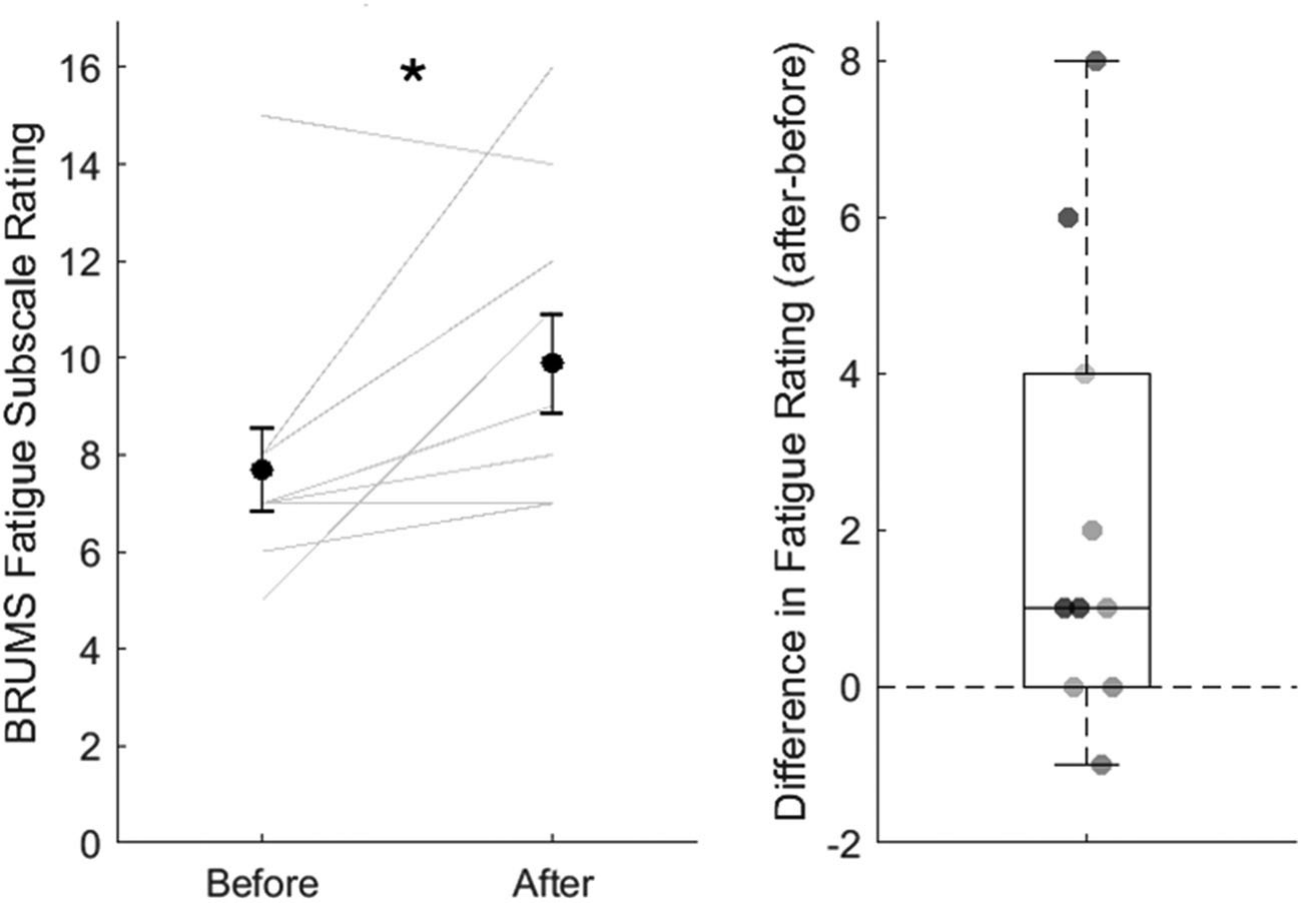
BRUMS fatigue scores. (a) Before and after completing the cognitive fatigue task battery. Black circles represent the mean score. Error bars show ±1 standard error. Light grey lines show individual scores. (b) Difference before and after completing the cognitive fatigue task battery. Values above the dotted line represent an increase in fatigue. Statistical significance shown: *P < 0.05. Unpublished data from Corfield and Davies (2024).

In the original study (Hassan et al., 2024), each cognitive task was performed for 10 min at a time and was repeated three times, resulting in a total duration of 2 h (four tasks × 10 min per task × three repeats = 4 × 10 min × 3 = 120 min). In the present study we will reduce the duration of each task to 2 min, giving a total duration of 24 min (four tasks × 2 min per task × three repeats = 4 × 2 min × 3 = 24 min). This has been done in consultation with our patient and public involvement group to avoid placing excessive burden on a population that reports high fatigue levels. The modified duration is expected to induce fatigue as participants are required to task switch between multiple short‐duration tasks, constituting an additional demand that increases cognitive fatigue (Dang et al., 2013). Testing of this modified task in 15 young healthy individuals indicates a significant increase in fatigue from pre‐ to post‐task. Fatigue measured on a 100 mm visual analogue scale (VAS) increased from a mean (standard error) of 26 (5) mm pre‐task to 59 (5) mm post‐task (Student's paired *t*‐test *t* = −7.251; *P* < 0.001). Fatigue measured using the fatigue subscale of the Brunel Mood Scale increased from a median (interquartile range) of 7.0 (1.5) pre‐task to 9.0 (4.0) post‐task (Wilcoxon signed rank test *Z* = 2.991; *P* = 0.003) (Corfield and Davies, unpublished data; Figures 3 and 4). Participants will be sent a training version of this protocol prior to attending the lab session, and this will allow them to familiarize themselves with the tasks involved to minimize any learning effect during the testing session. The cognitive task will be completed on a computer that will be placed in front of participants. Full details of the task are in Supporting information, Appendix 2.

#### 3.3.2 Physical task

The physical task is the 6‐minute walk task (6MWT). This requires participants to walk at their fastest pace for 6 min, aiming to cover as much ground as possible. Participants are able to take breaks at any point, with the clock continuously running (American Thoracic Society, 2002).

The test will be carried out on a self‐paced treadmill (Motek Medical). Four retroreflective markers will be placed on the pelvis over the left and right anterior and posterior superior iliac spines. The position of these markers will be recorded by a three‐dimensional optoelectronic motion capture system (Vicon Nexus, Oxford, UK) and streamed in real time into D‐flow software version 3.36.2 (Motek Medical). D‐flow software will monitor the position of these markers and control treadmill speed according to the position of the participant. This allows the participant to walk on the treadmill at a fluctuating speed, including slowing to a stop for rests whenever they want. The speed of the treadmill belt and the distance covered will be recorded within D‐flow software. Participants will be fitted with a security harness and will be allowed to familiarize themselves with the self‐paced treadmill for up to 5 min prior to the start of the physical task. Participants will be allowed to hold onto the siderails for stability.

The 6MWT induced fatigue in individuals with multiple sclerosis (McLoughlin et al., 2016), indicating it is an appropriate task to induce fatigue in long COVID participants. It is not anticipated that this task will cause significant fatigue in healthy (control) participants. However, healthy (control) participants will be asked to perform the task to provide control data and inform interpretation of data from participants with long COVID fatigue.

### 3.4 Outcome measures

#### 3.4.1 Participant characteristics

Participant characteristics (age, sex, ethnicity) will be captured using a custom form (Supporting information, Appendix 1). Trait fatigue (severity and impact), emotional symptoms, illness beliefs, and autonomic nervous system symptoms will be evaluated using the validated measures outlined below. This will allow appropriate descriptive reporting of the characteristics of the study sample and will also be used in exploratory analyses to develop future hypotheses (Research Question 4).

The severity of trait fatigue will be evaluated using the Fatigue Assessment Scale (FAS) and the Chalder Fatigue Questionnaire (CFQ‐11). The impact of fatigue will be evaluated using the Modified Fatigue Impact Scale (MFIS). The FAS is a validated unidimensional measure of fatigue that evaluates fatigue severity (Michielsen et al., 2003). The FAS consists of 10 items, each on a 5‐point rating scale, with total score ranging from 10 to 50. The FAS provides one unidimensional score for fatigue, with higher scores indicating greater fatigue severity. This measure is recommended as a core outcome measure for long COVID fatigue (Gorst et al., 2023), and inclusion will allow for comparison across long COVID research. The CFQ‐11 (Chalder et al., 1993) and MFIS (Ritvo et al., 1997) are multidimensional measures that provide individual scores for individual dimensions of fatigue. The CFQ‐11 consists of 11 items, each on a 3‐point Likert scale. The CFQ‐11 produces two individual scores of mental and physical fatigue. The MFIS consists of 21 items, which provide three individual scores of the perceived impact of physical, cognitive and psychosocial fatigue.

Self‐reported autonomic system function will be evaluated using the Composite Autonomic Symptom Score‐31 (COMPASS‐31) scale, which evaluates neurodegenerative system symptoms through 31 patient‐reported questions (Sletten et al., 2012). Assessment is through six weighted domains: orthostatic intolerance (10 points), vasomotor (6 points), secretomotor (7 points), gastrointestinal (28 points), bladder (9 points) and pupillomotor (15 points). A higher score indicates higher severity of autonomic dysfunction. The COMPASS‐31 scale has been used to measure autonomic dysfunction in long COVID populations. A large survey of individuals with long COVID found that 66% of patients were classified as having moderate to severe autonomic dysfunction quantified as a COMPASS‐31 score of ≥20 (Larsen et al., 2022).

Mood will be evaluated using the Hospital Anxiety and Depression Scale (HADS) (Zigmond & Snaith, 1983), which is a reliable and valid measure for assessing anxiety and depression (Herrmann, 1997). The HADS comprises 14 items, seven relating to anxiety and seven to depression. Items are rated on a 4‐point rating scale and produce two scores, one for anxiety (HADS‐A) and one for depression (HADS‐D). Scores can be interpreted as non‐cases (<7), mild (8–10), moderate (11–14) and severe (15–21) symptoms (Zigmond & Snaith, 1983).

Illness beliefs will be evaluated using the Brief Illness Perception Questionnaire (B‐IPQ) (Broadbent et al., 2006). The B‐IPQ is an eight‐item questionnaire that assesses cognitive illness representations, emotional illness representations, and illness coherence representation. The B‐IPQ has evidence of test–retest reliability and concurrent and discriminant validity (Broadbent et al., 2006). Each item is rated on a 0−10 scale, with higher scores indicating a more threatening perception of the illness. The total score is calculated by summing the scores of all eight items, with a possible range of 0–80. Higher scores indicate distorted or unhelpful illness beliefs.

#### 3.4.2 State fatigue

State fatigue will be evaluated using a 100 mm VAS that asks participants to rate their current level of fatigue. The VAS will be anchored on the left side with ‘not fatigued at all’ and on the right side with ‘extremely fatigued’ (Supporting information, Appendix 3).

Participants will be asked to make a line on a paper copy of the VAS at six time points across each testing session: at baseline (BL), immediately after the pre‐task somatosensory gating measure (PRE‐1), immediately after the pre‐task somatosensory attenuation measure (PRE‐2), immediately after the completion of the task (cognitive or physical) (POST‐1), immediately after the post‐task somatosensory gating measure (POST‐2), and immediately after the post‐task somatosensory attenuation measure (POST‐3) (Table 1 and Figure 2). This is to allow evaluation of the time course of fatigue and recovery of fatigue over the period required for the outcome measures. State fatigue at each time point will be quantified as the distance of the mark made by the participant from the left‐edge of the horizontal line (range 0−100 mm).

**TABLE 1:** Outcome measures.

		Time point of measurement		
Concept	Measure	Prior to first testing session	Cognitive task testing session	Physical task testing session
Severity of trait fatigue	FAS CFQ‐11 total CFQ‐11 physical subscale CFQ‐11 mental subscale	Prior to session		
Impact of trait fatigue	MFIS physical subscale MFIS cognitive subscale MFIS psychosocial subscale	Prior to session		
Autonomic symptoms	COMPASS‐31	Prior to session		
Mood	HADS‐A HADS‐D	Prior to session		
Illness beliefs	BIPQ	Prior to session		
Autonomic function	HRV		During somatosensory attenuation measure and throughout cognitive and physical task	During somatosensory attenuation measure and throughout cognitive and physical task
State fatigue	Visual analogue scale		BL, PRE‐1, PRE‐2, POST‐1, POST‐2, POST‐3	BL, PRE‐1, PRE‐2, POST‐1, POST‐2, POST‐3
State fatigue	Numeric rating scale		After every fourth task and at the end of the cognitive task	90 s, 180 s and 270 s and at the end of the 6MWT
Perceived effort	Rating of perceived exertion scale		After every fourth task and at the end of the cognitive task	90 s, 180 s and 270 s and at the end of the 6MWT
Cognitive fatigability	BIS		During cognitive task	
Physical fatigability	Average walking speed from 330 to 360 s of 6MWT minus average walking speed from 30 s to 60 s			During physical task
Somatosensory attenuation	Mean force overcompensation at each target force Intercept and slope from a linear regression of matched versus target force		Pre‐ and post‐task	Pre‐ and post‐task
Somatosensory gating	*I*_50_rest_ − *I*_50_movement_		Pre‐ and post‐task	Pre‐ and post‐task

Abbreviations: BIPQ, Brief Illness Perception Questionnaire; BIS, Balanced Integration Score; BL, Baseline; CFQ‐11, Chalder Fatigue Questionnaire; COMPASS‐31, Composite Autonomic Symptom Score; FAS, Fatigue Assessment Scale; HADS‐A, Hospital Anxiety and Depression Scale anxiety subscale; HADS‐D, Hospital Anxiety and Depression Scale depression subscale; HRV, heart rate variability; MFIS, Modified Fatigue Impact Scale; POST‐1, immediately after the completion of the task (cognitive or physical); POST‐2, immediately after the post‐task somatosensory attenuation measure; POST‐3, immediately after the post‐task somatosensory gating measure; PRE‐1, immediately after pre‐task somatosensory attenuation measure; PRE‐2, immediately after pre‐task somatosensory gating measure.

In addition, state fatigue will be reported using a numerical rating scale (NRS) at four time point during the physical and cognitive tasks. This will be at 90, 180 and 270 s of the 6MWT and after every fourth task in the cognitive task and at the end of each task. In the cognitive task these ratings will be time stamped. Participants will be shown the NRS, which consists of a scale from 0 to 10, with 0 being anchored as ‘not fatigued at all’ and 10 being anchored as ‘extremely fatigued’. They will be asked to verbally report the number that corresponds to their fatigue level. The need to switch from a VAS to an NRS is dictated by the inability of participants to physically mark a line on a piece of paper while completing the 6MWT.

#### 3.4.3 Perceived effort

Perceived effort will be evaluated using the Borg rating of perceived exertion (RPE) scale. This requires participants to rate how much effort an activity takes on a scale of 6 (no exertion at all) to 20 (maximal exertion). RPE will be measured at the same time points as the NRS. Participants will be shown the RPE scale prior to carrying out the tasks, as done in previous studies where participants were asked to report their RPE during a task (Flairty & Scheadler, 2020). This will allow us to make distinctions between perceived effort and fatigue during exertion. Participants will be shown the RPE scale during each task, and will be asked to verbally report the number that corresponds to their level of exertion.

#### 3.4.4 Autonomic nervous system function

Autonomic nervous system function will be quantified using heart rate variability (HRV), calculated as the proportion of successive intervals which differ by >50 ms (Baker et al., 2023). Heart rate will be measured using a polar H10 Hartslagsensor (Polar) streamed into Dflow software (Motek Medical). HRV will be measured during the somatosensory attenuation measure to ensure that participants are sitting quietly, not engaged in trial activity. This will replicate previous measurement in individuals with long COVID to allow comparison (Baker et al., 2023). HRV will additionally be measured throughout the cognitive and physical tasks to allow exploratory analysis of the change in autonomic nervous system function during exertion. HRV measured over a short period mostly reflects parasympathetic nervous system activity (Gullett et al., 2023). HRV has been used as a measure of autonomic nervous system function to quantify autonomic function changes in response to various interventions (Ali & Chen, 2023). Example code used to calculate HRV is provided as Supporting information.

#### 3.4.5 Fatigability during the cognitive task

Fatigability during the cognitive task (Research Question 2) will be quantified as reported by Hassan et al. (2024) using the balanced integration score (BIS) (Liesefeld & Janczyk, 2019). The AX‐CPT was chosen as the task in which to measure task performance, and therefore it will be presented first and last, following the protocol of Hassan et al. (2024). The BIS will be calculated using the reaction time and response data during the first and last repeat of the AX‐CPT task. The BIS combines reaction time and accuracy into a single metric which is standardized across all time points and participants. A BIS score of zero represents an average level of performance across all participants and conditions, with above average and below average performance indicated by positive and negative numbers, respectively. The code used to calculate the outcome measure is available at https://github.com/Liesefeld/BIS.

#### 3.4.6 Fatigability during the physical task

Fatigability during the 6MWT (Research Question 2) will be quantified as the change in average walking speed (m/s) from the beginning (30−60 s) to the end (330−360 s) of the test (Andersen et al., 2016; Witherspoon et al., 2018). The first time point will be taken after the initial 30 s to avoid confounding from acceleration. Example data and the code used to calculate fatigability in during the 6MWT are provided as Supporting information.

#### 3.4.7 Somatosensory processing

Two measures of somatosensory processing will be evaluated at each of the pre‐ and post‐task time points: a measure of somatosensory attenuation and a measure of somatosensory gating.

##### Somatosensory attenuation

The somatosensory attenuation assessment is expected to take 10 min. Participants will be seated comfortably and positioned with their dominant hand placed on a table with their palm facing upwards. Participants will be given a practice trial to make sure they understand the task.

An audible beep will mark the start of the trial. The researcher (B.T.) will press on the abductor pollicis brevis muscle of the dominant hand with a Digital Handheld Dynamometer (microFET2; Hoggan Scientific, Salt Lake City, UT, USA). The force will be applied through a digit transducer pad of 1 cm diameter to a magnitude of 4, 5, 6 or 7 N, and will be maintained for 3 s until a second beep marks the end of the 3 s. This constitutes an exafferent force. The magnitude of force applied is displayed in real time on the device and will be visible to the researcher, but not to the participant. After 2 s rest, a third beep will sound, and the participant will be asked to press the same probe onto the same section of skin with the same force that was exerted by the researcher with a second Digital Handheld Dynamometer (microFET2, Hoggan Scientific). This constitutes a reafferent force (Kilteni & Ehrsson, 2022).

Following a practice trial, each force level (4, 5, 6 or 7 N) will be performed eight times, for a total of 32 trials. These trials will be organized in two blocks of 16 trails. The force level will be randomized within MATLAB software. There will be an interval of 60 s between the two blocks. The average force across the 3 s is stored in the dynamometer for each trial and will be retrieved after the end of the session.

Somatosensory attenuation will be quantified as the mean force overcompensation between the exafferent force (generated by the researcher) and the reafferent force (generated by the participant). Mean force overcompensation will be calculated as the average difference between the exafferent and reafferent force (Wolpe et al., 2018), on each trial (*n* = 8) across each force level (*n* = 4). To examine force matching as a function of force level, the intercept and slope from the linear regression between exafferent and reafferent force will be calculated for each participant and condition. Example data are shown in Supporting information Figure S3, and the code used to calculate the outcome measure is provided as Supporting information.

##### Somatosensory gating

The protocol to evaluate somatosensory gating replicates that of Baker et al. (2023) to allow direct comparison of results and is expected to take 20 min. The participant will be seated comfortably and positioned with their dominant hand placed on a table.

The index finger of the dominant hand will be stimulated with single, constant current, square wave pulses of 2 ms duration by a constant current stimulator (model DS7A; Digitimer; Welwyn Garden City, UK) connected to adhesive surface electrodes (71505‐K/C/12; Ambu Ltd, Alconbury Weald, UK) placed on the proximal and middle phalanges.

Stimulation will be mild, starting below perceptual threshold and rising to the threshold at which it can just be perceived, but never above this. At this threshold, the stimulation feels like a mild transient ‘tingle’. Perceptual threshold will be determined prior to the task by asking participants to report verbally when they feel a stimulation on their index finger. The initial stimulus intensity will be 0.10 mA. Based on previous work (Chapman et al., 1987), we expect this to be imperceptible to all participants. The intensity will be adjusted using an ascending–descending–ascending staircase method as follows:

- A set of up to six stimuli will be delivered at the initial intensity. If the participant does not report perceiving a stimulus on at least three out of six trials, stimulation intensity will be increased by 0.10 mA for the next set. This continues until the participant reports that they perceive a stimulus on at least three out of six trials.
- At this point, the stimulus intensity is then decreased by 0.05 mA per set until the participant does not report perceiving a stimulus on three out of six trials.
- The stimulation intensity is then increased by 0.01 mA per set until the participant again reports that they perceive a stimulus on three out of six trials. This value is recorded as the perceptual threshold.

Based on previous work (Baker et al., 2023; Chapman et al., 1987) it is anticipated that perceptual threshold will range from 0.23 to 1.11 mA across participants.

One hundred trials will then be performed, during which participants will be given a stimulus (*P* = 0.8) or not (*P* = 0.2). Fifty of these will be ‘resting’ trials, and 50 will be movement trials. In a resting trial, participants will be asked if they are ready, and asked to report if they felt a stimulation or not. In a movement trial, participants will be asked if they are ready, and then instructed to make a rapid index finger abduction movement and report if they felt a stimulation or not. Stimulation will have been delivered in 80% of the trials. Activity of the first dorsal interosseus muscle will be monitored using surface electromyography (Delsys Trigno Quattro Sensor) and monitored in real time in D‐flow software (Motek Medical). The software will be programmed to detect when the muscle activity starts to increase above baseline and will deliver a stimulus (*P* = 0.8) or not (*P* = 0.2) 50 ms after this onset of muscle activity. In this way, the timing of the stimulus in the movement trials is directly linked to movement onset. In all trials, participants will be given up to 5 s to respond if they felt stimulus or not. If they are unable to respond, this will be reported as stimulus not perceived.

Rest and movement trails (*n* = 50 of each) will be randomized. Stimulus intensity will start at the pre‐determined perceptual threshold and will subsequently be controlled in equal steps separately for rest and movement trials (Baker et al., 2023). If the participant perceives the stimulus, it will be decreased by 0.02 mA for the next trial in that condition. If the participant does not perceive the stimulus, it will be increased by 0.02 mA for the next trial in that condition. There will be an inter‐stimulus interval of at least 3 s between consecutive trials.

Somatosensory gating will be quantified in the same way as reported by Baker et al. (2023), using code provided by these authors. The probability of detection at intensity *I* will be fitted to a sigmoid curve:

$$P\left(\mathrm{Detection}\;\mathrm{at}\;\mathrm{intensity}\;I|I_{50},k\right)=\frac{1}{1+exp[\left(I_{50}-I\right)/k)]}$$

where *I*_50_ is the intensity with 50% detection, and *k* determines the slope of the curve. This will be quantified in rest and movement trials, and somatosensory gating is *I*_50_movement_ − *I*_50_rest_. Example sigmoid curves calculated at rest and during movement are shown in Supporting information, Figure S4.

### 3.5 Data inclusion and exclusion

Participants failing to complete the cognitive task will be excluded from the analysis for Research Question 2, 3 and 4 as we will not be able to compare the impact of task. However, their data will be included in other exploratory analysis where the cognitive task is not included as a predictor. Participants who do not complete the post‐task measures in either session, due to withdrawing from the study, will be excluded from the analysis for Research Question 3.

### 3.6 Data analysis

Participant characteristics (age, sex, ethnicity) will be described using measures of frequency distribution. All other outcomes (FAS score, CFQ‐11 total and subscale scores, MFIS total and subscale scores, COMPASS‐31 score, HADS score, B‐IPQ score, BIS score, change in walking speed, HRV during quiet sitting in each session, HRV throughout each task (physical, cognitive), VAS score at six time points in each session, NRS at four time points during each task, RPE at four time points during each task, somatosensory attenuation pre‐ and post‐task in each session, somatosensory gating pre‐ and post‐task in each session) will be described using measures of central tendency and distribution. A list of all outcomes is provided in Table 1.

### 3.7 Statistical analysis

A series of statistical analyses will be conducted to compare the descriptive characteristics of the two groups (long COVID/neither long COVID nor fatigue). The characteristics to be compared are age, gender, trait fatigue (FAS score, CFQ‐11 total score, CFQ‐11 physical subscale score, CFQ‐11 mental subscale score), impact of trait fatigue (MFIS physical subscale score, MFIS cognitive subscale score, MFIS psychosocial subscale score), mood (HADS‐A score, HADS‐D score), illness beliefs (BIPQ score), and autonomic symptoms (COMPASS‐31 score). Gender will be analysed as a categorical variable. A chi‐square test will be used to compare the gender distribution between the groups. All other variables will be analysed as ratio data and compared across groups using an independent samples if the data meet parametric assumptions (normality, verifying equal variance) and a Mann–Whitney *U* test if the data do not meet parametric assumptions.

Results will be summarized using a measure of central tendency (mean or median) and spread (standard deviation or interquartile range), test statistics, and *P*‐values for ratio data and frequency, percentage, chi‐square values, and *P*‐values for categorical variables.

Hypothesis 1 Pre‐task (baseline) somatosensory attenuation will be different between individuals with long COVID fatigue and controls. We will also attempt to replicate the Baker et al. (2023) finding that somatosensory gating is not different between individuals with long COVID fatigue and controls. The independent variable will be ‘participants’, which consists of two categories: ‘individuals with long COVID fatigue’ and ‘controls’. The two dependent variables will be ‘somatosensory attenuation’ and ‘somatosensory gating’, measured at baseline during the first session participants attend.

This will be tested using a multivariate analysis of variance to test for an overall difference in somatosensory processing between populations, and to explore the differences in somatosensory gating and attenuation. We will check that the following assumptions are met: multivariate normality, independence and equal variance. If we find the multivariate analysis of variance is significant, we will conduct follow‐up univariate analysis of variance for somatosensory attenuation and somatosensory gating to identify specific contributions to the effect and will apply Bonferroni correction to control for the increased risk of Type I error.

Hypothesis 2 : State fatigue following exertion will be predicted by somatosensory attenuation at baseline, somatosensory gating at baseline, the presence of long COVID (long covid/controls), and the type of task (cognitive/physical). Performance fatigability will be considered a moderator variable in these relations. State fatigue prior to exertion will be included as a covariate in this analysis.

This will be tested using a linear mixed model:

$$Y_{ijk}=\mu +a_{i}+b_{j}+c_{k}+d_{ik}+e_{ik}+f_{ik}+g_{ijk}+\left(f_{ik}\times a_{i}\right)+\left(f_{ik}\times b_{j}\right)+\varepsilon_{ijk}$$

where:

- *Y_ijk_*: state fatigue following exertion in the *i*‐th group (long COVID/control) during task *j* (cognitive/physical) for the *k*‐th subject.
- μ: the overall mean of state fatigue following exertion.
- *a_i_*: the effect of the *i*‐th group (fixed effect: long COVID vs. control).
- *b_j_*: the effect of the *j*‐th task (fixed effect: cognitive vs. physical).
- *d_ik_*: the baseline somatosensory attenuation for the *k*‐th subject in the *i*‐th group.
- *e_ik_*: the baseline somatosensory gating for the *k*‐th subject in the *i‐*th group.
- *f_ijk_*: the performance fatigability for the *k*‐th subject in the *i*‐th group during the *j*‐th task.
- *g_ijk_*: the state fatigue prior to exertion for the *k*‐th subject in the *i*‐th group during the *j*‐th task (covariate).
- (*f_ik_* ×*a_i_*): the interaction term between performance fatigability and group.
- (*f_ik_* ×*b_j_*): the interaction term between performance fatigability and task.
- *ϵ_ijk_*: the residual error term, assumed to be normally distributed.

Hypothesis 3 : The change in somatosensory attenuation (Research Question 3a) and somatosensory gating (Research Question 3b) from pre‐ to post‐task will be predicted by the change in state fatigue from pre to post, fatigability, the presence of long COVID (long COVID/controls), and the type of task (cognitive/physical).

We will use linear mixed models to test these hypotheses, with separate models for somatosensory attenuation and somatosensory gating:

$$Y_{ijk}=a_{i}+b_{j}+c_{ijk}+d_{ijk}+u_{0i}+\varepsilon_{ijk}$$

where:

- *Y_ijk_*: attenuation or gating at baseline in the *i*‐th group (long COVID/control) during task j (cognitive/physical) for the k‐th subject.
- *a_i_*: the fixed effect of the *i*‐th group (long COVID vs. control) on baseline attenuation.
- *b_j_*: the fixed effect of the *j*‐th task (cognitive vs. physical) on baseline attenuation.
- *c_ijk_*: the change in state fatigue from pre to post in the *i*‐th group, during task *j*, for the k‐th subject, affecting baseline attenuation.
- *d_ijk_*: the change in fatigability in the *i*‐th group, during task *j*, for the *k*‐th subject,
- *u*_0_ *_i_*: random intercept for group *i*, capturing group‐level variability
- *ϵ_ijk_*: residual error term, representing random variability and measurement error not explained by the fixed effects.

Hypothesis 4 : Among individuals with long COVID, trait fatigue will be effected by mood, illness beliefs, autonomic symptoms, increase in state fatigue induced by a physical task, the increase in state fatigue induced by a cognitive task, and autonomic nervous system function.

This will be tested using a linear mixed effects model:

$$Y_{i}=\mu +a_{i}+b_{i}+c_{i}+d_{i}+e_{i}+f_{i}+u_{0i}+\varepsilon_{i}$$

where:

- *Y_i_*: trait fatigue for individual *i*.
- μ: the overall mean of state fatigue following exertion.
- *a_i_*: mood for individual *i*.
- *b_i_*: illness beliefs for individual *i*.
- *c_i_*: autonomic symptoms for individual *i*.
- *d_i_*: change in state fatigue induced by physical task for individual *i*.
- *e_i_*: change in state fatigue induced by cognitive task for individual *i*.
- *f_i_*: autonomic nervous system function for individual *i*
- *u*_0_ *_i_*: random intercept capturing individual‐specific variability in trait fatigue not explained by the fixed effects.
- *ϵ_i_*: residual error term representing random variability and measurement error.

We will check the following assumptions of the regression models: linearity, independence, homoscedasticity, normality and multicollinearity.

#### 3.7.1 Planned exploratory analysis

- Hypotheses 1, 2, 3 will be rerun with age included as a covariate.
- Hypotheses 1, 2, 3, 4 will be rerun with time of day (morning/afternoon) at which participants completed sessions and session order (cognitive/physical first) included as additional predictor variables.
- The effect of autonomic dysfunction during exertion, on state fatigue pre‐ to post‐exertion, via an effect on somatosensory processing post‐exertion will be tested using mediation analysis with separate models for somatosensory attenuation and somatosensory gating. This will be quantified in participants with and without long COVID.
- The relations between autonomic dysfunction during exertion, state fatigue during exertion, and perceived effort during exertion will be quantified in participants with and without long COVID during both tasks (cognitive/physical). This will be tested using correlation analysis.
- Test‐retest reliability will be conducted for each somatosensory processing measure (gating and attenuation), from the baseline measure from each session (cognitive/physical). This will provide a measure of whether somatosensory processing is stable over time.

We will carry out outcome neutral criteria that must be met for successful testing of the stated hypothesis. This includes checking for the absence of floor or ceiling effects. This will be done by examining data distributions to ensure that there are no extreme values that could impact the validity of results. Additionally, we will investigate the quality of the data and this will include frequency measures and the shape of the distribution.

### 3.8 Sample size

The study is powered to address Research Question 1a. Previous research indicates an effect size of 0.78 for differences in somatosensory attenuation between young and older individuals (Parthasharathy et al., 2022). Powering the current study to detect an effect size of 0.78 between two independent means for Research Question 1a—somatosensory attenuation in individuals with and without long COVID—with an α error probability of 0.05 and a power of 95% and based on a non‐directional two‐tailed hypothesis requires 44 participants per group (G*power 3.1.9.7). Dropout is not expected to impact Research Question 1a or 1b, as it involves initial measurements during the first session. However, there is a potential for dropout to impact the subsequent research questions, which rely on participants returning for a second session.

The sample size gives us power of 90% to detect an effect of 0.70 between two groups at two repeated time points with alpha error probability of 0.05 for Research Questions 2 and 3, or a power of 80% to detect an effect of 0.60. A dropout rate of 10% would mean that our power to detect these effect sizes decreases to 60%. The approach for addressing Research Questions 2 and 3 ensures that the study is adequately powered to detect correlations of 0.50 and 0.40, respectively. The code used to calculate the outcome measure is available at https://github.com/smancuso/R‐ Code/blob/cc90c623c4dc733fe5c93ef5a795dde010152c63/Power.R

### 3.9 Timeline

July 2024–April 2025: recruitment and data collection.

April 2025–May 2025: processing of data.

May 2025–June 2025: collating of results.

June 2025–August 2025: submission of Stage 2 registered report.

## AUTHOR CONTRIBUTIONS

All piloting was performed in the facilities at Cardiff University. Bethan Thomas, Rachael Pattinson, Christine Bundy, and Jennifer L. Davies contributed to the conception and design of the study. Bethan Thomas, Rachael Pattinson, Christine Bundy, and Jennifer L. Davies were responsible for acquisition, analysis, or interpretation of data for the work. Bethan Thomas, Rachael Pattinson, Christine Bundy, and Jennifer L. Davies drafted the work and revised it critically for important intellectual content. All authors have approved the final version of the manuscript, and all authors agree to be accountable for all aspects of the work in ensuring that questions related to the accuracy or integrity of any part of the work are appropriately investigated and resolved. All persons designated as authors qualify for authorship, and all those who qualify for authorship are listed.

## CONFLICT OF INTEREST

The authors declare no conflicts of interest.

## Supporting information

Appendix 1. Screening questions.

Appendix 2. Cognitive task.

Appendix 3. Visual analogue scale.

Data collected during piloting for the sensory attenuation task.

Figure 3. Example somatosensory attenuation results.
Figure 4. Example somatosensory gating results.

Code for the sensory attenuation task, to analyse sensory attenuation force data and produce outcomes.

Data collected from piloting during the 6MWT.

Code for the 6MWT to analyse and produce 6MWT outcomes.

HRV code to analyse HR during piloting and produce HRV outcome.

## References

[eph13615-bib-0001] Ali, M. K. , & Chen, J. D. Z. (2023). Roles of heart rate variability in assessing autonomic nervous system in functional gastrointestinal disorders: A systematic review. Diagnostics, 13(2), 293. 36673103 10.3390/diagnostics13020293 PMC9857852

[eph13615-bib-0002] American Thoracic Society . (2002). ATS statement: Guidelines for the six‐minute walk test. American Journal of Respiratory and Critical Care Medicine, 166(1), 111–117. 12091180 10.1164/ajrccm.166.1.at1102

[eph13615-bib-0003] Andersen, L. K. , Knak, K. L. , Witting, N. , & Vissing, J. (2016). Two‐ and 6‐minute walk tests assess walking capability equally in neuromuscular diseases. Neurology, 86(5), 442–445. 26740680 10.1212/WNL.0000000000002332

[eph13615-bib-0004] Bailey, A. , Channon, S. , & Beaumont, J. G. (2007). The relationship between subjective fatigue and cognitive fatigue in advanced multiple sclerosis. Multiple Sclerosis, 13(1), 73–80. 17294614 10.1177/1352458506071162

[eph13615-bib-0005] Baker, A. M. E. , Maffitt, N. J. , del Vecchio, A. , McKeating, K. M. , Baker, M. R. , Baker, S. N. , & Soteropoulos, D. S. (2023). Neural dysregulation in post‐COVID fatigue. Brain Communications, 5(3), fcad122. 37304792 10.1093/braincomms/fcad122 PMC10257363

[eph13615-bib-0006] Broadbent, E. , Petrie, K. J. , Main, J. , & Weinman, J. (2006). The brief illness perception questionnaire. Journal of Psychosomatic Research, 60(6), 631–637. 16731240 10.1016/j.jpsychores.2005.10.020

[eph13615-bib-0007] Callan, C. , Ladds, E. , Husain, L. , Pattinson, K. , & Greenhalgh, T. (2022). ‘I can't cope with multiple inputs’: A qualitative study of the lived experience of ‘brain fog’ after COVID‐19 [Research Support, Non‐U.S. Gov't]. British Medical Journal Open, 12(2), e056366. 10.1136/bmjopen-2021-056366 PMC8844964 35149572

[eph13615-bib-0008] Chalder, T. , Berelowitz, G. , Pawlikowska, T. , Watts, L. , Wessely, S. , Wright, D. , & Wallace, E. P. (1993). Development of a fatigue scale. Journal of Psychosomatic Research, 37(2), 147–153. 8463991 10.1016/0022-3999(93)90081-p

[eph13615-bib-0009] Chapman, C. E. , Bushnell, M. C. , Miron, D. , Duncan, G. H. , & Lund, J. P. (1987). Sensory perception during movement in man. Experimental Brain Research, 68(3), 516–524. 3691723 10.1007/BF00249795

[eph13615-bib-0010] Clark, J. E. , & Watson, S. (2023). Modelling mood updating: A proof of principle study. The British Journal of Psychiatry, 222(3), 125–134. 36511113 10.1192/bjp.2022.175 PMC9929713

[eph13615-bib-0011] Costa, T. , Taylor, A. , Black, F. , Hill, S. , McAllister‐Williams, R. H. , Gallagher, P. , & Watson, S. (2023). Autonomic dysregulation, cognition and fatigue in people with depression and in active and healthy controls: Observational cohort study. The British Journal of Psychiatry Open, 9(4), e106. 37313995 10.1192/bjo.2023.68 PMC10305035

[eph13615-bib-0012] Crook, H. , Raza, S. , Nowell, J. , Young, M. , & Edison, P. (2021). Long covid‐mechanisms, risk factors, and management [Review]. British Medical Journal, 374, n1648. 34312178 10.1136/bmj.n1648

[eph13615-bib-0013] Dang, J. , Dewitte, S. , Mao, L. , Xiao, S. , & Shi, Y. (2013). Adapting to an initial self‐regulatory task cancels the ego depletion effect. Consciousness and Cognition, 22(3), 816–821. 23742871 10.1016/j.concog.2013.05.005

[eph13615-bib-0014] de Doncker, W. , Brown, K. E. , & Kuppuswamy, A. (2021). Influence of post‐stroke fatigue on reaction times and corticospinal excitability during movement preparation. Clinical Neurophysiology, 132(1), 191–199. 33302061 10.1016/j.clinph.2020.11.012 PMC7810236

[eph13615-bib-0015] de Doncker, W. , Dantzer, R. , Ormstad, H. , & Kuppuswamy, A. (2018). Mechanisms of poststroke fatigue. Journal of Neurology, Neurosurgery and Psychiatry, 89(3), 287–293. 28939684 10.1136/jnnp-2017-316007

[eph13615-bib-0016] Dotan, A. , David, P. , Arnheim, D. , & Shoenfeld, Y. (2022). The autonomic aspects of the post‐COVID19 syndrome. Autoimmunity Reviews, 21(5), 103071. 35182777 10.1016/j.autrev.2022.103071 PMC8848724

[eph13615-bib-0017] Enoka, R. M. , Almuklass, A. M. , Alenazy, M. , Alvarez, E. , & Duchateau, J. (2021). Distinguishing between fatigue and fatigability in multiple sclerosis. Neurorehabilitation and Neural Repair, 35(11), 960–973. 34583577 10.1177/15459683211046257

[eph13615-bib-0018] Fancourt, D. , Steptoe, A. , & Bu, F. (2023). Psychological consequences of long COVID: Comparing trajectories of depressive and anxiety symptoms before and after contracting SARS‐CoV‐2 between matched long‐ and short‐COVID groups. British Journal of Psychiatry, 222(2), 74–81. 10.1192/bjp.2022.155 PMC7614126 36458509

[eph13615-bib-0019] Fietsam, A. C. , Bryant, A. D. , & Rudroff, T. (2023). Fatigue and perceived fatigability, not objective fatigability, are prevalent in people with post‐COVID‐19. Experimental Brain Research, 241(1), 211–219. 36462035 10.1007/s00221-022-06518-0 PMC9735153

[eph13615-bib-0020] Flairty, J. E. , & Scheadler, C. M. (2020). Perceived and heart rate‐based intensities during self‐paced walking: Magnitudes and comparison. International Journal of Exercise Science, 13(5), 677–688. 32509131 10.70252/LUDF2243 PMC7241645

[eph13615-bib-0021] Gorst, S. L. , Seylanova, N. , Dodd, S. R. , Harman, N. L. , O'Hara, M. , Terwee, C. B. , Williamson, P. R. , Needham, D. M. , Munblit, D. , & Nicholson, T. R. , PC‐COS study group . (2023). Core outcome measurement instruments for use in clinical and research settings for adults with post‐COVID‐19 condition: An international Delphi consensus study. Lancet Respiratory Medicine, 11(12), 1101–1114. 37926103 10.1016/S2213-2600(23)00370-3

[eph13615-bib-0022] Gullett, N. , Zajkowska, Z. , Walsh, A. , Harper, R. , & Mondelli, V. (2023). Heart rate variability (HRV) as a way to understand associations between the autonomic nervous system (ANS) and affective states: A critical review of the literature. International Journal of Psychophysiology, 192, 35–42. 37543289 10.1016/j.ijpsycho.2023.08.001

[eph13615-bib-0024] Hassan, E. K. , Jones, A. M. , & Buckingham, G. (2024). A novel protocol to induce mental fatigue. Behavior Research Methods, 56(4), 3995–4008. 37537491 10.3758/s13428-023-02191-5 PMC11133042

[eph13615-bib-0025] Herrmann, C. (1997). International experiences with the hospital anxiety and depression scale–a review of validation data and clinical results. Journal of Psychosomatic Research, 42(1), 17–41. 9055211 10.1016/s0022-3999(96)00216-4

[eph13615-bib-0026] Ito, S. , Kimura, T. , & Gomi, H. (2022). Attribution of sensory prediction error to perception of muscle fatigue. Scientific Reports, 12(1), 16708. 36202958 10.1038/s41598-022-20765-9 PMC9537327

[eph13615-bib-0027] Jamali, A. , Sadeghi‐Demneh, E. , Fereshtenajad, N. , & Hillier, S. (2017). Somatosensory impairment and its association with balance limitation in people with multiple sclerosis. Gait & Posture, 57, 224–229. 28667904 10.1016/j.gaitpost.2017.06.020

[eph13615-bib-0028] Jammoul, M. , Naddour, J. , Madi, A. , Reslan, M. A. , Hatoum, F. , Zeineddine, J. , Abou‐Kheir, W. , & Lawand, N. (2023). Investigating the possible mechanisms of autonomic dysfunction post‐COVID‐19. Autonomic Neuroscience, 245, 103071. 36580747 10.1016/j.autneu.2022.103071 PMC9789535

[eph13615-bib-0029] Joli, J. , Buck, P. , Zipfel, S. , & Stengel, A. (2022). Post‐COVID‐19 fatigue: A systematic review. Frontiers in Psychiatry, 13, 947973. 36032234 10.3389/fpsyt.2022.947973 PMC9403611

[eph13615-bib-0030] Kessner, S. S. , Bingel, U. , & Thomalla, G. (2016). Somatosensory deficits after stroke: A scoping review. Topics in Stroke Rehabilitation, 23(2), 136–146. 27078117 10.1080/10749357.2015.1116822

[eph13615-bib-0031] Kilteni, K. , & Ehrsson, H. H. (2022). Predictive attenuation of touch and tactile gating are distinct perceptual phenomena. Iscience, 25(4), 104077. 35372807 10.1016/j.isci.2022.104077 PMC8968059

[eph13615-bib-0032] Kluger, B. M. , Krupp, L. B. , & Enoka, R. M. (2013). Fatigue and fatigability in neurologic illnesses: Proposal for a unified taxonomy. Neurology, 80(4), 409–416. 23339207 10.1212/WNL.0b013e31827f07be PMC3589241

[eph13615-bib-0033] Krupp, L. B. , & Elkins, L. E. (2000). Fatigue and declines in cognitive functioning in multiple sclerosis. Neurology, 55(7), 934–939. 11061247 10.1212/wnl.55.7.934

[eph13615-bib-0034] Kuppuswamy, A. (2017). The fatigue conundrum. Brain, 140(8), 2240–2245. 28899013 10.1093/brain/awx153 PMC5806506

[eph13615-bib-0035] Kuppuswamy, A. (2022). The neurobiology of pathological fatigue: New models, new questions. The Neuroscientist, 28(3), 238–253. 33446049 10.1177/1073858420985447 PMC9136477

[eph13615-bib-0036] Larsen, N. W. , Stiles, L. E. , Shaik, R. , Schneider, L. , Muppidi, S. , Tsui, C. T. , Geng, L. N. , Bonilla, H. , & Miglis, M. G. (2022). Characterization of autonomic symptom burden in long COVID: A global survey of 2,314 adults. Frontiers in Neurology, 13, 1012668. 36353127 10.3389/fneur.2022.1012668 PMC9639503

[eph13615-bib-0037] Leventhal, H. , Phillips, L. A. , & Burns, E. (2016). The Common‐Sense Model of Self‐Regulation (CSM): A dynamic framework for understanding illness self‐management. Journal of Behavioral Medicine, 39(6), 935–946. 27515801 10.1007/s10865-016-9782-2

[eph13615-bib-0038] Liesefeld, H. R. , & Janczyk, M. (2019). Combining speed and accuracy to control for speed‐accuracy trade‐offs(?). Behavior Research Methods, 51(1), 40–60. 30022459 10.3758/s13428-018-1076-x

[eph13615-bib-0039] Lou, J. S. , Kearns, G. , Benice, T. , Oken, B. , Sexton, G. , & Nutt, J. (2003). Levodopa improves physical fatigue in Parkinson's disease: A double‐blind, placebo‐controlled, crossover study. Movement Disorders, 18(10), 1108–1114. 14534913 10.1002/mds.10505

[eph13615-bib-0040] Matura, L. A. , Malone, S. , Jaime‐Lara, R. , & Riegel, B. (2018). A systematic review of biological mechanisms of fatigue in chronic illness. Biological Research for Nursing, 20(4), 410–421. 29540066 10.1177/1099800418764326 PMC6346311

[eph13615-bib-0041] Maxwell, L. J. , Jones, C. , Bingham, C. O. , Boers, M. , Boonen, A. , Choy, E. , Christensen, R. , Conaghan, P. G. , D'Agostino, M. A. , Doria, A. S. , Grosskleg, S. , Hill, C. L. , Hofstetter, C. , Horgan, B. , Kroon, F. , Leung, Y. Y. , Mackie, S. , Meara, A. , Shea, B. J. , … Beaton, D. E. (2024). Defining domains: Developing consensus‐based definitions for foundational domains in OMERACT core outcome sets. Seminars in Arthritis and Rheumatism, 66, 152423. 38460282 10.1016/j.semarthrit.2024.152423

[eph13615-bib-0042] McLoughlin, J. V. , Barr, C. J. , Patritti, B. , Crotty, M. , Lord, S. R. , & Sturnieks, D. L. (2016). Fatigue induced changes to kinematic and kinetic gait parameters following six minutes of walking in people with multiple sclerosis. Disability and Rehabilitation, 38(6), 535–543. 25990573 10.3109/09638288.2015.1047969

[eph13615-bib-0043] Michielsen, H. J. , de Vries, J. , & van Heck, G. L. (2003). Psychometric qualities of a brief self‐rated fatigue measure: The fatigue assessment scale. Journal of Psychosomatic Research, 54(4), 345–352. 12670612 10.1016/s0022-3999(02)00392-6

[eph13615-bib-0044] National Institute for Health and Care Excellence . (2020). COVID‐19 rapid guideline *: managing the long‐term effects of COVID‐19*. https://www.nice.org.uk/guidance/ng188 33555768

[eph13615-bib-0046] Parthasharathy, M. , Mantini, D. , & Orban de Xivry, J. J. (2022). Increased upper‐limb sensory attenuation with age. Journal of Neurophysiology, 127(2), 474–492. 34936521 10.1152/jn.00558.2020

[eph13615-bib-0047] Ritvo, P. , Fischer, J. S. , Miller, D. M. , Andrews, H. , Paty, D. W. , & LaRocca, N. G. (1997). Multiple sclerosis quality of life inventory: A user's manual. https://nms2cdn.azureedge.net/cmssite/nationalmssociety/media/msnationalfiles/brochures/msqli_‐a‐user‐s‐manual.pdf 10.1177/135245859900500410 10467384

[eph13615-bib-0048] Sletten, D. M. , Suarez, G. A. , Low, P. A. , Mandrekar, J. , & Singer, W. (2012). COMPASS 31: A refined and abbreviated composite autonomic symptom score. Mayo Clinic Proceedings, 87(12), 1196–1201. 23218087 10.1016/j.mayocp.2012.10.013 PMC3541923

[eph13615-bib-0049] Thomas, B. , Pattinson, R. , Edwards, D. , Dale, C. , Jenkins, B. , Lande, H. , Bundy, C. , & Davies, J. L. (2024). Defining and measuring long COVID fatigue—a scoping review [Unpublished raw data]. School of Healthcare Sciences, Cardiff University. 10.1136/bmjopen-2024-088530 PMC11647363 39663173

[eph13615-bib-0050] Timar, L. , Job, X. , Orban de Xivry, J. J. , & Kilteni, K. (2023). Aging exerts a limited influence on the perception of self‐generated and externally generated touch. Journal of Neurophysiology, 130(4), 871–882. 37609705 10.1152/jn.00145.2023 PMC10642979

[eph13615-bib-0051] Townsend, L. , Dyer, A. H. , Jones, K. , Dunne, J. , Mooney, A. , Gaffney, F. , O'Connor, L. , Leavy, D. , O'Brien, K. , Dowds, J. , Sugrue, J. A. , Hopkins, D. , Martin‐Loeches, I. , Ni Cheallaigh, C. , Nadarajan, P. , McLaughlin, A. M. , Bourke, N. M. , Bergin, C. , O'Farrelly, C. , … Conlon, N. (2020). Persistent fatigue following SARS‐CoV‐2 infection is common and independent of severity of initial infection. PLoS ONE, 15(11), e0240784–e0240784. 33166287 10.1371/journal.pone.0240784 PMC7652254

[eph13615-bib-0052] Trott, M. , Driscoll, R. , & Pardhan, S. (2022). The prevalence of sensory changes in post‐COVID syndrome: A systematic review and meta‐analysis. Frontiers in Medicine, 9, 980253. 36091707 10.3389/fmed.2022.980253 PMC9452774

[eph13615-bib-0053] Witherspoon, J. W. , Vasavada, R. P. , Melissa, R. W. , Shelton, M. , Chrismer, I. C. , Wakim, P. G. , Jain, M. S. , Bonnemann, C. G. , & Meileur, K. G. (2018). 6‐minute walk test as a measure of disease progression and fatigability in a cohort of individuals with RYR1‐related myopathies. Orphanet Journal of Rare Diseases, 13(1), 105. 29970108 10.1186/s13023-018-0848-9 PMC6029052

[eph13615-bib-0054] Wolpe, N. , Zhang, J. , Nombela, C. , Ingram, J. N. , Wolpert, D. M. , Cam, C. A. N. , & Rowe, J. B. (2018). Sensory attenuation in Parkinson's disease is related to disease severity and dopamine dose. Scientific Reports, 8(1), 15643. 30353104 10.1038/s41598-018-33678-3 PMC6199336

[eph13615-bib-0055] Zigmond, A. S. , & Snaith, R. P. (1983). The hospital anxiety and depression scale. Acta Psychiatrica Scandinavica, 67(6), 361–370. 6880820 10.1111/j.1600-0447.1983.tb09716.x

[eph13615-bib-0056] Zijdewind, I. , Kernell, D. , & Kukulka, C. G. (1995). Spatial differences in fatigue‐associated electromyographic behaviour of the human first dorsal interosseus muscle. The Journal of Physiology, 483(Pt 2), 499–509. 7650617 10.1113/jphysiol.1995.sp020601 PMC1157860

