# Supplementary material for: Somatosensory processing in long COVID fatigue and its relations with physiological and psychological factors
Source: Exp Physiol. 2024 Aug 6;109(10):1637–49. doi: 10.1113/EP091988 (PMC11442760; doi:10.1113/EP091988)
Supplement: Supplementary file 1 — Appendix 1. Screening questions. [file EPH-109-1637-s007.docx]

# Appendix 1 – Screening questions

These will be completed by the participants online (as described in Figure 2). The online form is available here - https://app.onlinesurveys.jisc.ac.uk/s/cardiff/somatosensory-processing-in-long-covid-fatigue-and-its-relati-1 The questions contained in the form are shown below.

*Disclosure of the information on this form is voluntary. This information is collected to ensure that it is safe for you to participate in the study, and for reporting purposes. The information you provide in this form is confidential and will not be shared with anyone outside of the research team.*

**Participants will be asked to complete the following questions using a drop down box of provided options:**

- Age

- Sex

- Ethnicity

**Participants will be asked to indicate whether they have a past or current history of the following question. If they respond yes, they will be given space to provide more information:**

- Covid-19 infection

**If participants answered yes to the previous question, they will be asked the following questions:**

- How were you diagnosed with COVID 19? (tick one option - polymerase chain reaction test, positive lateral flow, clinical diagnosis, other)
- Within the past 12 months have you been hospitalised as a result of COVID-19 infection? (tick box of yes or no)
- Have you experienced signs and symptoms that have continued or developed 12 weeks or more after your initial acute COVID 19 infection? (tick box of yes or no)
- Is fatigue one of the symptoms you have continued to experience following COVID-19 infection? – Fatigue is considered a range of symptoms from mild subjective feelings of tiredness to an overwhelming debilitating, and sustained sense of exhaustion that likely decreases one's ability to execute daily activities and function normally in family or social roles. This can include any form of fatigue (physical, cognitive, mental, psychosocial, post exertional malaise). (tick box of yes or no)
- History of Present Condition (inc. Severity of initial infection, aggravating and easing factors). (describe in space provided)
- Duration of long COVID (estimated date of when long COVID symptoms started).

**All participants will be asked to respond yes or no to the following questions:**

- Do you experience chronic or recurring fatigue? - this can include any form of fatigue (physical, cognitive, mental, psychosocial, post exertional malaise)
- Do you have any injury, chronic illness or other medical condition that interferes with daily function?
- Do you have difficulty with physical exercise, or advice from a physician not to exercise (now or within the past three months)?
- Within the past 12 months have you had an orthopaedic problem, traumatic injury or surgery?
- Are you, or could you be pregnant?
