# Supplementary material for: Somatosensory processing in long COVID fatigue and its relations with physiological and psychological factors
Source: Exp Physiol. 2024 Aug 6;109(10):1637–49. doi: 10.1113/EP091988 (PMC11442760; doi:10.1113/EP091988)
Supplement: Supplementary file 3 — Appendix 3. Visual analogue scale. [file EPH-109-1637-s008.docx]

# Appendix 3 – Visual analogue scale

Fatigue is considered a range of symptoms from mild subjective feelings of tiredness to an overwhelming debilitating, and sustained sense of exhaustion that likely decreases one's ability to execute daily activities and function normally in family or social roles. This can include any form of fatigue (physical, cognitive, mental, psychosocial, post exertional malaise).

These will be completed by the participants in both testing sessions on multiple occasions (as described in Figure 2).

**How would you rate your fatigue in this current moment?**

Not physically/cognitively fatigued at all

Extremely physically/cognitively fatigued
