# Supplementary material for: Somatosensory processing in long COVID fatigue and its relations with physiological and psychological factors
Source: Exp Physiol. 2024 Aug 6;109(10):1637–49. doi: 10.1113/EP091988 (PMC11442760; doi:10.1113/EP091988)
Supplement: Supplementary file 5 — Figure 3. Example somatosensory attenuation results. Figure 4. Example somatosensory gating results. [file EPH-109-1637-s002.docx]

###
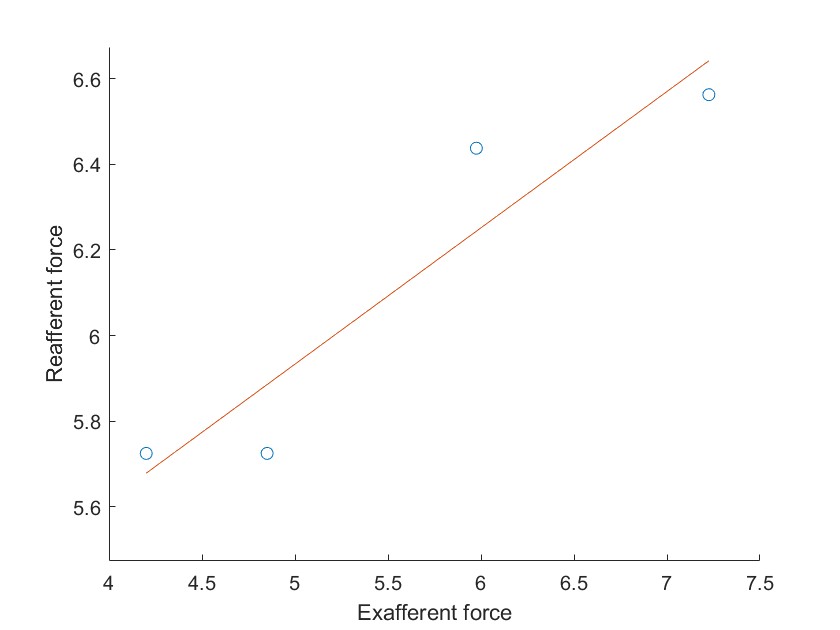


Figure 3 – Example somatosensory attenuation results. Data points show the mean exafference (exerted by researcher) and reafference (exerted by participant) force across eight trials at each of four target force levels. Line shows linear regression (intercept, slope). Intercept = 4.342. Slope = 0.318


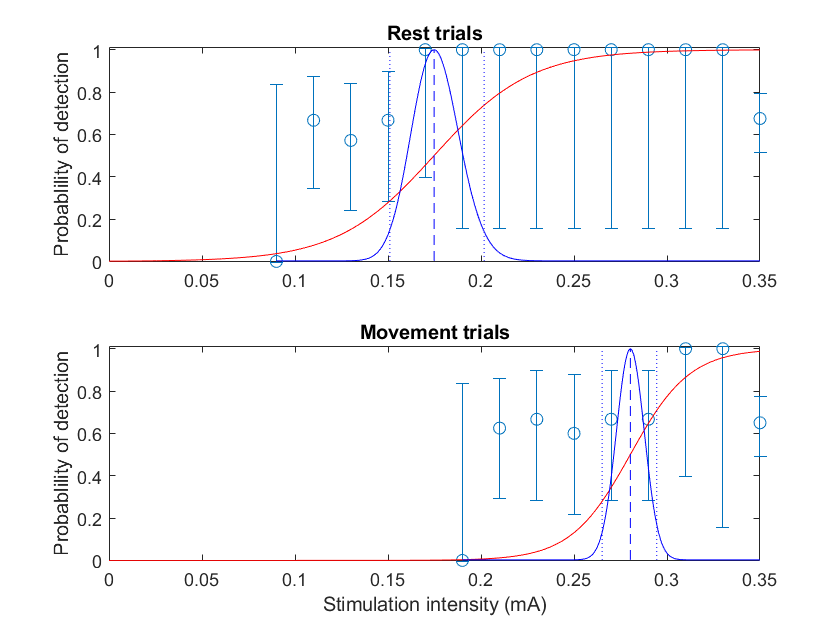


Figure 4 – Example somatosensory gating results. Top panel shows probability of detection during rest trials and bottom panel shows probability of detection during movement trials. Open circles indicate probability of detection at each intensity, error bars represent 95% confidence intervals. Red line shows sigmoid curve fitted to these data. Blue vertical dashed line indicates I_50_ (0.175 mA for rest and 0.280 mA for movement in this example). Somatosensory gating in this example is 0.280 – 0.175 = 0.105. Code to fit sigmoid curve to data and calculate I_50_ was provided by M. Baker and is that used in Baker et al. (2023).
